# Comparative Evaluation of the Cyclic Fatigue Resistance of WaveOne Gold in Reciprocation, ProGlider in Rotary Motion, and Manual Files in a Reciprocating Handpiece Within Simulated Curved Canals: An In Vitro Study

**DOI:** 10.7759/cureus.67704

**Published:** 2024-08-24

**Authors:** Shivangi M Pujara, Hardik B Shah, Leena H Jobanputra

**Affiliations:** 1 Department of Conservative Dentistry and Endodontics, Government Dental College and Hospital, Jamnagar, Jamnagar, IND

**Keywords:** waveone gold, endodontics, glidepath, reciprocation, cyclic fatigue resistance

## Abstract

Aim

This study compares the cyclic fatigue resistance of glide path files versus manual files used with a reciprocating handpiece in simulated curved canals, specifically evaluating WaveOne Gold Glider, ProGlider, stainless steel (S.S.) K files, nickel-titanium (NiTi) K files, and Flexo K files.

Materials and methods

Seventy-five instruments were divided into five groups of 15. Simulated canals with a 45° angle and a 5 mm radius of curvature were created using laser micromachining on S.S. blocks. A reciprocating endodontic handpiece (TEP-ER10, NSK, Japan) was used for the manual files and an X-Smart Plus endo motor for the rotary files. The time to fracture was recorded via videography. Statistical analysis was performed using analysis of variance (ANOVA) and post-hoc Tukey tests.

Results

Flexo files exhibited the highest mean cyclic fatigue resistance (1690.33 ± 456.97 seconds), followed by WaveOne Gold Glider (872.13 ± 210.08 seconds), NiTi K files (760.73 ± 163.86 seconds), S.S. K files (707.60 ± 257.98 seconds), and ProGlider (371.00 ± 66.02 seconds). Fragment length differences were statistically insignificant among the groups. ANOVA showed significant differences in cyclic fatigue resistance but not in fragment length.

Conclusion

Flexo files showed superior cyclic fatigue resistance, making them ideal for glide path formation. NiTi and S.S. K files with a reciprocating handpiece performed similarly to WaveOne Gold Glider, providing a cost-effective alternative to rotary systems, especially in resource-limited areas. ProGlider had the lowest resistance, highlighting the advantage of reciprocating motion in enhancing durability.

## Introduction

The concept of a glide path encompasses a smoothly contoured radicular pathway extending from the coronal opening to the radiographic apical terminus or the terminus indicated by electronic apex locators [1]. This pathway is an essential precursor for facilitating the precise navigation of rotary endodontic instruments into the canal. Establishing a well-defined glide path is recommended before the insertion of larger-sized rotary files for shaping. Typically, this initial root canal preparation is executed utilizing small-sized and slightly tapered nickel-titanium (NiTi) rotary or stainless-steel (S.S.) K files [2].

While certain experts advocate for the utilization of hand files in this process, highlighting their capacity to offer tactile feedback and minimize procedural risks, it is acknowledged that manual preparation can prove laborious and time-consuming [3-4].

An alternative approach to gain the benefits of hand files while overcoming their drawbacks involves their mechanized utilization with reciprocating handpieces. Reciprocating handpieces are designed for the mechanical preparation of root canals, using manual endodontic instruments in a forward and backward motion with preset angles specific to the handpiece. These devices need to be connected to an air motor for operation [5]. They have emerged as valuable adjuncts in endodontics, offering enhanced operator comfort, reduced operative time, and decreased operator fatigue. For instance, the Nakanishi TEP E10R (NSK Inc., Tokyo, Japan) exemplifies automated systems for root canal preparation, delivering heightened efficiency and ergonomic advantages [6].

The NSK TEP ER10 handpiece is an air-driven, 10:1 gear reduction handpiece that generates a reciprocating motion with 60 degrees of movement in both clockwise and counterclockwise directions, mimicking manual hand motion. It supports a maximum speed of 40,000 rotations per minute (rpm) and is compatible with hand files. It does not have an autoreverse function.

Recent advancements in technology have yielded nickel-titanium (NiTi) instruments with superior alloys like heat-treated alloy, controlled memory, R phase, and reciprocation purportedly augmenting their resistance to cyclic fatigue. This advancement has led to the development of several new NiTi rotary systems characterized by a reduced number of instruments, simplifying the instrumentation process [7].

Notably, ProGlider, a novel single-file rotary pathfinding system crafted from heat-treated M-wire alloy by Dentsply Maillefer, features a progressive taper ranging from 2% to 8% along its length, accompanied by four cutting edges and a square cross-section [8].

Another noteworthy addition to the landscape of NiTi instruments is the WaveOne Gold Glider (Dentsply Sirona, Ballaigues, Switzerland), meticulously fabricated using NiTi subjected to multiple heat treatment and cooling cycles to enhance its flexibility and strength. It has a parallelogram-shaped cross-section (15 tip diameter) and exhibits taper increments ranging from 2% to over 6% across its active segment.

Despite the surge in the popularity of NiTi rotary or reciprocating instruments among practitioners and endodontic specialists, 2% S.S.K and NiTi manual files continue to enjoy widespread usage for the initial negotiation of canals, facilitating the establishment of an endodontic glide path [9].

However, the adoption of rotary systems is impeded in some regions [10], particularly developing countries, due to the high cost associated with NiTi rotary or reciprocating files. By contrast, reciprocating handpieces are employed in conjunction with conventional files, thereby reducing operational expenses compared to continuous rotary or reciprocating instrumentation.

Cyclic fatigue refers to the progressive structural degradation of a material when subjected to repeated cyclic loading, leading to the development of microcracks and ultimately culminating in mechanical failure. Cyclic fatigue occurs as the instrument rotates or reciprocates within the curved and narrow confines of the root canal, experiencing alternating tensile and compressive stresses. Over time, these cyclic stresses can cause the material to undergo fatigue-induced degradation, compromising its integrity and increasing the risk of fracture [11-12] While reciprocation with NiTi instruments has become very popular in recent years, with a significant number of research and published articles [13-14], only a few have been written in the last decades about reciprocation with conventional instruments [15]. Moreover, none of these articles was specifically designed to evaluate the possible use of reciprocation and conventional hand files in the creation of a glide path. Since contemporary studies have shown that reciprocation can improve the fatigue resistance of NiTi instruments [16], it is interesting to evaluate if, and to which extent, hand K files can gain from a reciprocating motion during the creation of an endodontic glide path.

The study aimed to compare the cyclic fatigue resistances of WaveOne Gold Glider, Proglider, S.S. K files, NiTi K files, and Flexo K files with reciprocating handpiece in simulated canals having 45° angle of curvature and a 5 mm radius of curvature.

The null hypothesis was that there is no significant difference in the cyclic fatigue resistance among the WaveOne Gold Glider files, ProGlider files, and S.S. K files, NiTi K files, and Flexo K files mounted on the reciprocating handpiece.

## Materials and methods

Using G*Power 3.1.9.4 (Universitat Kiel, Germany) for analysis, the sample size was calculated with a 95% confidence level (α = 4%) and 80% power (β = 20%). Given these parameters, the minimum required sample size for each group was determined to be 15 instruments. Therefore, 15 instruments were assigned to each group.

In Group 1 (WaveOne Gold Glider (WOG) group), the WOG file features a tip size of 0.15 and variable tapers that increase from 2% to over 6% along its active portion (Dentsply Sirona); in Group 2 (ProGlider (PG) group), the PG NiTi rotary file (tip size of 0.16 tip with progressive taper 2% near tip to 8% near the shaft) (Dentsply Sirona, Ballaigues, Switzerland); in Group 3 (S.S. group), S.S. K file (tip size 0.15 tip with constant taper 2%) (Mani, Inc. Japan); in Group 4 (NITIFLEX group), NITIFLEX K file (tip size 0.15 tip with constant taper 2%) (Dentsply Maillefer, Switzerland); and in Group 5 (K-Flexo group), K-Flexo S.S. file (tip size 0.15 tip with constant taper 2%) (Dentsply Maillefer, Switzerland).

Before the experimental procedure, each file was inspected for defects and deformities using a Semorr 3000D surgical microscope (Germany) at 20x magnification.

Preparation of simulated canals

Artificial canals were machined in a stainless-steel plate of dimensions 55 mm x 35 mm x 5 mm, by utilizing the laser micromachining technology [17]. An industrial fiber laser (ZLine, India) was used for machining. The artificial canal was designed using Corel Draw, and the laser path was programmed with a Standard Triangle Metals language file specific to the proprietary machine program. The laser operated at a wavelength of 1064 nm with a peak power of 30 watts.

The laser was concentrated on the metal piece using the Galvano scanner after the establishment of laser process parameters, and then the canal was machined layer by layer [17]. In an S.S. block, the artificial canals were machined with similar dimensions to the specifications of the file examined to avoid the file binding in the testing apparatus. Canals were prepared 0.1 mm wider and 0.2 mm deeper than the file size, allowing for some lateral movement within the canal. During the procedure, the block was covered with a transparent glass of 4 mm thickness with clips to provide a closed chamber.

**Figure 1:**
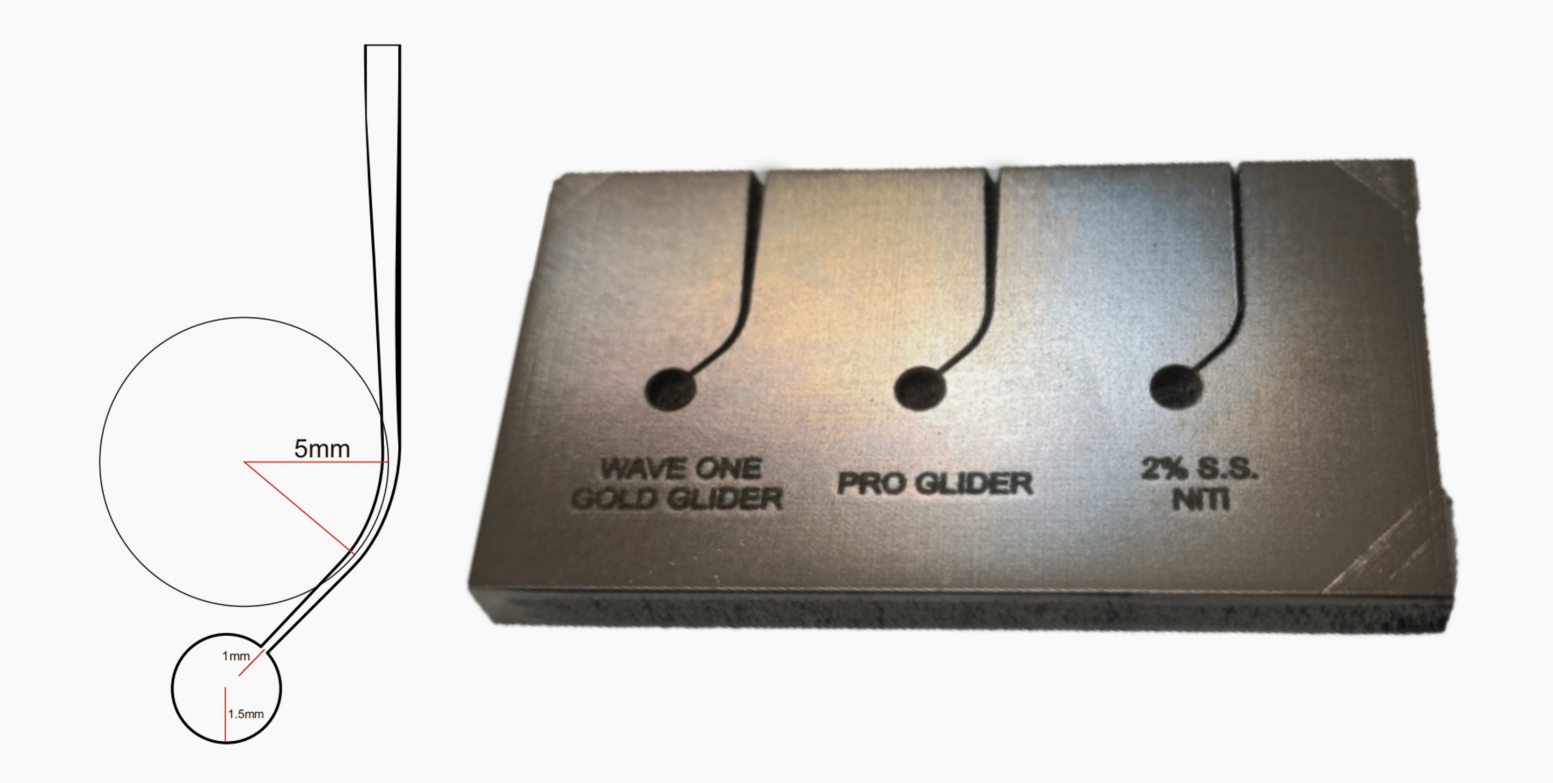
Design of the simulated canals and laser-machined stainless-steel block for WaveOne Gold Glider, Pro Glider and 2% S.S., NiTi, and Flexo files

To determine the dimensions of the artificial canals, a straight line was drawn along the long axis of the canal's coronal section. A second line was drawn at a 45-degree angle along the long axis of the canal's apical section. Each line intersected at points where the canal curvature began (point a) and ended (point b). The curving segment of the canal was represented by a circle with tangents at points a and b. The angle of curvature was measured as the number of degrees on the arc of the circle between points a and b, with a radius of 5 mm and a curvature center located 5 mm from the instrument tip [18]. An additional circle of diameter 3 mm, overlapping 1 mm of the tip of the file, was laser-machined to prevent binding of the file in the apical part. Individual markings of each group were made onto the metal block (Figure 1).

Cyclic fatigue testing

**Figure 2:**
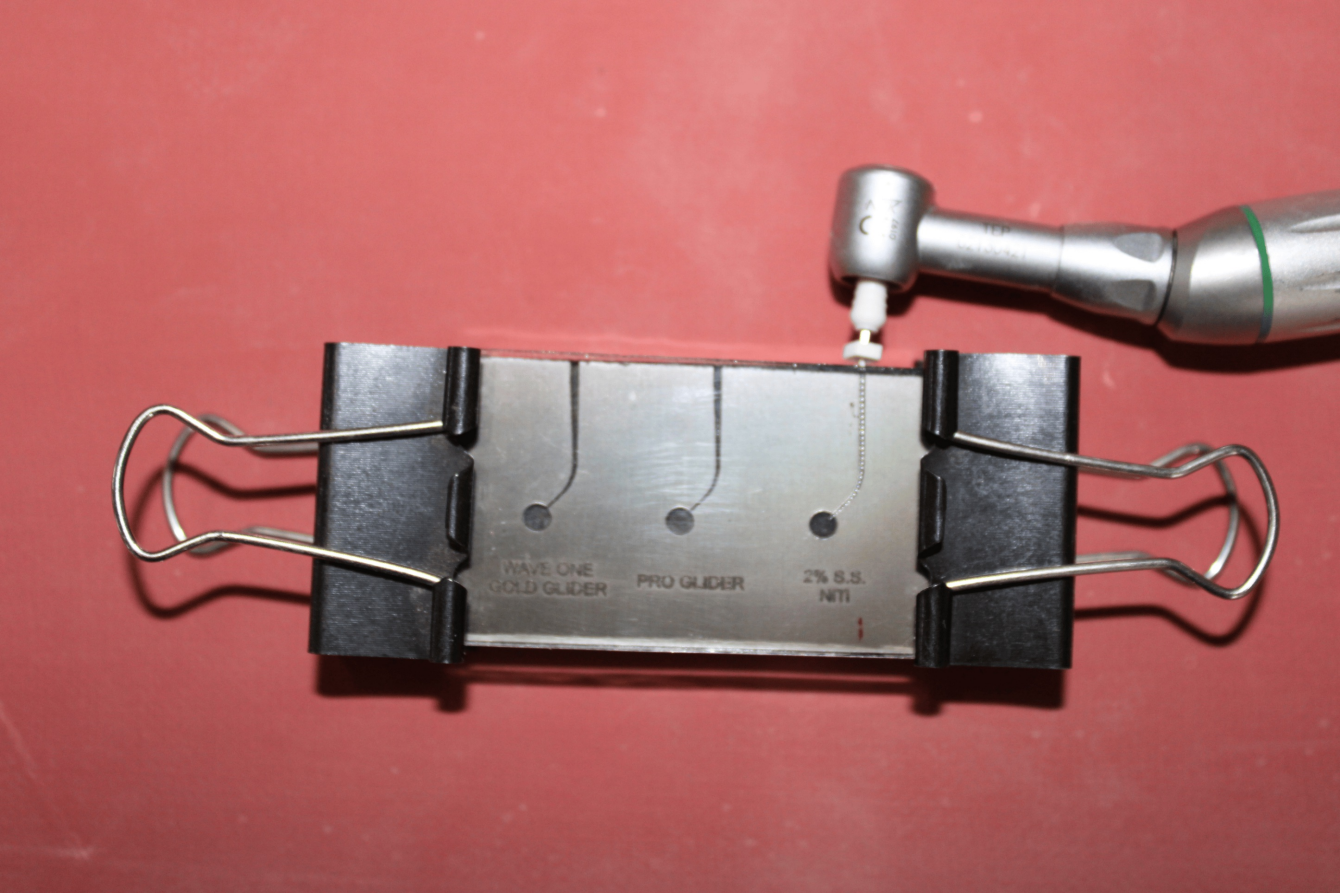
File with handpiece inside the simulated canal for cyclic fatigue testing

All rotary files were powered by X-Smart Plus endo motor (Dentsply, Sirona) at a controllable torque set according to the manufacturer’s operating specifications: ProGlider (PG) used a rotary motion at a speed of 300 rpm with a torque of 2 N/cm, while WaveOne Gold (WOG) operated with a reciprocating motion, “WaveOne All” mode. All hand files were powered by NSK TEP ER 10 (Nakanishi, Japan) along with an air motor with calibrated speed in the range of 1800 to 2200 rpm (Figure 2). Glycerine was used as a lubricant during each test to minimize friction between the instrument and the device, as well as to reduce heat generation and dissipation [19].

**Figure 3:**
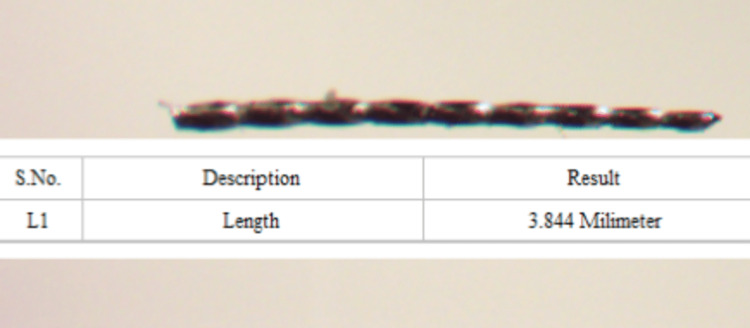
Fragment length measurement

The time to fracture was recorded via videography. The fragment length was measured under a LaboVision Z2000 stereomicroscope at 20× magnification for the distance from the instrument tip to the point of fracture (Figure 3).

For statistical analysis, data were tabulated, and a master chart was prepared in Microsoft Excel (Microsoft Corporation, USA). Then, data were analyzed with IBM SPSS (version 19.0, IBM Corporation, Armonk, NY, USA). One-way analysis of variance (ANOVA) with post-hoc Tukey test was performed to obtain significant differences among all the study groups. For all analyses, the significance level was set at 5%.

## Results

The mean and standard deviation of the cyclic fatigue resistance and fragment length for each instrument group are shown in Table 1. The intergroup comparison of the cyclic fatigue resistance test is shown in Table 2.

**Table 1:** Cyclic fatigue resistance and fragment length of different endodontic glide path instruments (n = 15 per system) Values are presented as mean ± standard deviation. P < 0.05. *One-way analysis of variance (ANOVA)

File system	Cyclic fatigue time (in seconds)	F value of ANOVA	Significance	Fragment length (in mm)	F value of ANOVA	Significance
WaveOne Gold	872.1333 ± 210.075	51.309	.000	3.721 ± 0.411	.258	.904
Dentsply ProGlider	371.000 ± 66.016			3.934 ± 0.567		
Stainless Steel 2%	707.600 ± 257.983			4.021 ± 0.336		
NiTiFLEX -K 2%	760.733 ± 163.858			3.932 ± 0.563		
K-Flexo 2%	1690.333 ± 456.973			3.940 ± 1.644		

**Table 2:** Intergroup comparison of the cyclic fatigue resistance test *HS = highly significant (p < 0.001), #S = significant (p < 0.05), NS = non-significant (p > 0.05)

Group	Comparison group	P-value after applying Tukey’s post-hoc test
WaveOne Gold	Proglider	0.000 ^ *^
	Stainless Steel	0.440
	NiTiFLEX-K	0.778
	K-Flexo	0.000 *
ProGlider	WaveOne Gold	0.000 *
	Stainless Steel	0.007 ^#^
	NiTiFLEX-K	0.001 *
	K-Flexo	0.000 *
Stainless Steel	WaveOne Gold	0.440
	Proglider	0.007 #
	NiTiFLEX-K	0.982
	K-Flexo	0.000 *
NiTiFLEX-K	WaveOne Gold	0.778
	Proglider	0.001 *
	Stainless Steel	0.982
	K-Flexo	0.000 *
K-Flexo	WaveOne Gold	0.000 *
	Proglider	0.000 *
	Stainless Steel	0.000 *
	NiTiFLEX-K	0.000 *

The lowest cyclic fatigue resistance was recorded in the ProGlider group (371.000 ± 66.016), followed by the S.S. group (707.600 ± 257.983), the NiTiFLEX- K group (760.733 ± 163.858), and the WaveOne Gold group (872.1333 ± 210.075), and the highest was observed in the K-Flexo group (1690.333 ± 456.973). The shortest mean fragment length was observed in the WaveOne Gold group (3.721 ± 0.411), followed by the NiTiFLEX-K group (3.932 ± 0.563), the ProGlider group (3.934 ± 0.567), the K-Flexo group (3.940 ± 1.644), and the longest in the S.S. group (4.021 ± 0.336). However, the difference was statistically insignificant (p < 0.05). The one-way ANOVA was significant for cyclic fatigue resistance and not significant for the fragment length. Hence, post-hoc Tukey was done only for cyclic fatigue resistance. The difference of the Flexo group was statistically highly significant with all the other groups (p < 0.001). WaveOne Gold, NiTiFLEX K, and S.S. groups showed statistically significant differences with the ProGlider and K-Flexo groups (p < 0.05), while the difference among them was statistically insignificant (p > 0.05). The difference of the ProGlider group was statistically significant with all the other groups (p < 0.05).

## Discussion

The introduction of the glide path concept has emphasized the necessity of reducing the occurrence of fractures in NiTi shaping instruments, particularly highlighting the role of glide path files in facilitating the safe application of noncutting and/or passive NiTi files. The establishment of a smooth pathway from the canal orifice to the apical foramen is crucial for improving endodontic treatment outcomes [20].

Unlike multisequence instrument systems that use two or three files, the instruments utilized in this study establish the glide path using only one, making them highly effective and convenient. A typical minimum glide path size is 0.15 mm apical preparation to ensure the safe introduction of the shaping instrument, which corresponds to the 15-tip size of the hand or rotary file. Consequently, for this study, a WaveOne Gold reciprocating file (tip size 15) and ProGlider (tip size 16), along with three hand files (each with a size 15) possessing approximately the same tip size, were selected [21].

Our study aimed to compare the cyclic fatigue resistance of commonly used 2% hand files made of different materials including stainless steel, NiTiFLEX-K, and K-Flexo files (constructed from flexible S.S.), when mechanized by mounting them on a reciprocating handpiece with a 60-degree reciprocating motion. This comparison was conducted against the rotary NiTi ProGlider, operated in rotation mode at a speed of 300 rpm and 2 N of torque as per the manufacturer's instructions, and the reciprocating WaveOne Gold system, using the WaveOne All preset program in a Dentsply X-Smart Plus endomotor. Evaluations were performed in a simulated 45-degree curved canal with dimensions corresponding to those of the individual file systems, following the in vitro model for cyclic fatigue testing as recommended by Pruett et al. [18].

It is essential to explore any potential synergistic benefits resulting from the combined usage of reciprocation movement and conventional hand files for glide path preparation. In addition, it is imperative to evaluate whether this combined approach is comparable to the utilization of rotary or reciprocating NiTi file systems. There have been studies using reciprocating handpieces with 30 degrees or 90 degrees of reciprocation [22-23]. To our knowledge, no studies have been done to evaluate cyclic fatigue resistance using a 60-degree handpiece. In the present study, the NSK TEP ER 10 handpiece was used to provide 60 degrees of reciprocation.

This study's findings indicate that despite the favorable mechanical properties of NiTi alloys and advancements in manufacturing NiTi instruments for endodontic use [24], reciprocating motion significantly influences the resistance to cyclic fatigue of endodontic instruments [25].

Endodontic files can experience both torsional and cyclic fatigue, which can cause the files to fracture. Torsional fatigue occurs when the file is engaged with the root canals while the handpiece continues rotating. Cyclic fatigue occurs when the file is rotated in a curved canal, causing repeated compressive and tensile stresses. The results of the present study for fragment length show that there was no significant difference among the groups. This signifies that the file fracture was because of the generation of cyclic fatigue at the site of maximum simultaneous tension and compression, and torsional fatigue did not affect the results of the study.

Flexo files exhibited the highest resistance to cyclic fatigue, as they are made up of flexible S.S. with a batt-tip, which avoids ledge formation by preventing gouging into the walls. These properties make it clinically suitable for glide path formation when used with reciprocation.

The WOG, NiTi, and S.S. groups did not show any significant difference. Many studies [26-27] have shown that WaveOne Gold has better cyclic fatigue resistance, owing to its reciprocation motion, but the interesting outcome of this study was that its performance was comparable to conventional S.S. and NiTi 2% 15 size K file when used with reciprocation, which are easily available and cost-effective.

Clinically, it will be easy to adapt, time-effective, economical, and comfortable for the practitioners already using these conventional files.

ProGlider showed the least cyclic fatigue resistance, highlighting the importance of reciprocation movement irrespective of the file system used [13].

The S.S. alloy is harder than NiTi, allowing for a more effective cutting action. Enhanced cutting efficiency and increased resistance to cyclic fatigue are crucial qualities for a glide path instrument designed to navigate and initially enlarge predominantly small, narrow, and curved canals [28]. Because only small S.S. K-files (up to ISO size 20) are utilized for creating a glide path, the rigidity of the stainless-steel alloy may pose a minimal drawback [29].

Furthermore, the forward-backward movement provided by reciprocation facilitated easier negotiation of curved canals and reduced the risk of tip blockage.

The 2% conventional NiTi file has shown similar results to WOG and 2% S.S., opening the option for clinicians to choose their preferred alloy with a reciprocating handpiece that gives the same result.

The tested instruments, PG and WOG, rotated at 300 rpm, whereas the K-file, being attached to a compressed air motor, operated at a higher speed. Research indicates that in cyclic fatigue tests, increasing the speed typically decreases the time to failure [30]

In this study, comparing or defining speed with consistent parameters such as rpm (rotations per minute) was not feasible due to the varied motions of the instruments. Hence, the results were quantified based on time to failure, a parameter more closely aligned with performance during intracanal use.

One study limitation is using simulated conditions, which may not fully replicate the complex and varied anatomical features found in natural teeth. In addition, the study focused on mechanical properties and cyclic fatigue resistance without considering potential biological factors or clinical outcomes. Furthermore, this study focussed only on cyclic fatigue and did not consider torsional fatigue, which the initial files used for glide path creation usually encounter in the narrow, unprepared canals. Finally, while the study explored reciprocating motion, it did not compare different degrees of reciprocation which could impact the findings and their applicability in clinical settings.

## Conclusions

The results indicate that employing a specific reciprocating motion, small-sized hand files made up of S.S. or NiTi instruments could be considered as an alternative method for establishing a glide path. Therefore, using a reciprocating handpiece for creating a mechanical endodontic glide path could be a valid choice.

Future clinical research should further explore the potential benefits of combining reciprocation with conventional hand files, providing clinicians with adaptable, time-efficient, and cost-effective options in clinical practice.
